# Differences between Sexes in Cardiopulmonary Fitness among Children and Adolescents with Kawasaki Disease

**DOI:** 10.3390/healthcare10020353

**Published:** 2022-02-11

**Authors:** Tzu-Hsuan Kuan, Yung-Liang Chang, Ko-Long Lin, Guan-Bo Chen, I-Hsiu Liou, Sheng-Hui Tuan

**Affiliations:** 1 Department of General Medicine, E-DA Hospital, Kaohsiung 824, Taiwan; sopugi@gmail.com; 2 Department of General Medicine, Kaohsiung Medical University Hospital, Kaohsiung 807, Taiwan; mil2589764@gmail.com; 3 Department of Pharmacy and Master Program, Ta-Jen University, Pingtung 907, Taiwan; kllinvghks@gmail.com (K.-L.L.); isliuvghks@gmail.com (I.-H.L.); 4 Department of Physical Medicine and Rehabilitation, Kaohsiung Veterans General Hospital, Kaohsiung 813, Taiwan; 5 Department of Internal Medicine, Kaohsiung Armed Forces General Hospital, Kaohsiung 802, Taiwan; tanwein@gmail.com; 6 Institute of Allied Health Sciences, National Cheng Kung University, Tainan 701, Taiwan; 7 Department of Rehabilitation Medicine, Cishan Hospital, Ministry of Health and Welfare, Kaohsiung 842, Taiwan

**Keywords:** Kawasaki disease, cardiopulmonary fitness, peak oxygen consumption, gender differences, self-efficacy

## Abstract

It is known that children and adolescents with Kawasaki disease (KD) can maintain normal cardiopulmonary fitness (CPF) after the disease’s acute stage has subsided. This study aimed to investigate whether gender differences affect CPF in children and adolescents with KD. We retrospectively reviewed a cohort of 204 participants (120 boys and 84 girls) with KD. All participants were instructed to complete a symptom-limited cardiopulmonary exercise test (CPET) adapted to assess their CPF. Based on body mass index (BMI), boys and girls were categorized into groups of underweight (19 boys and 12 girls), normal (62 boys and 59 girls), and overweight (39 boys and 13 girls). Although a similarity in body composition was found among both genders for KD subjects and normal Taiwanese peers, the percentage of overweight subjects was higher in KD boys than the normal Taiwanese boys. When comparing CPF for different BMI groups, the whole KD group showed no discrepancy, but a significantly lower peak VO2 for the overweight KD boys group was observed, representing poorer CPF. In conclusion, girls with KD had better CPF than boys, and gender stereotypes affect sports participation as well as self-efficacy, and may be contributing to poorer CPF in KD boys.

## 1. Introduction

Kawasaki disease (KD), first reported by Japanese pediatrician Tomisaku Kawasaki, is an acute but typically self-limiting vasculitis mostly occurring in children under the age of 5 years. The disease’s global incidence rate is particularly high in East Asia, with Japan reporting the highest incidence of 264.8 cases per 100,000 in 2012 and continuing to increase annually [1]. Following Japan and Korea, Taiwan is placed third with more than 50 cases per 100,000 people occurring yearly for children below the age of 5 [2]. Due to its unknown pathogenesis, the diagnosis is based on clinical criteria, including signs and symptoms such as fever, mucosal inflammation, peripheral extremity vascular changes, or cervical lymphadenopathy. As the disease primarily affects medium-sized muscular arteries, particularly the coronary arteries (CAs), it is the leading cause of acquired heart disease in children [3]. Therefore, early detection of KD is critical for timely intervention to significantly lower the risk of coronary artery lesions. Initial treatment with intravenous immunoglobulins (IVIG) and aspirin is the gold standard, which has proved to be effective in preventing the development of CA aneurysm, thereby, lowering the subsequent morbidity and mortality. When left untreated, CA abnormalities develop in about 25% of patients and lead to myocardial infarction, sudden death, or ischemic heart disease [4]. 

Notably, despite the risk of developing CA abnormalities, previous studies had suggested that KD patients have comparable levels of cardiopulmonary fitness (CPF) to their normal peers due to compromised coronary perfusion when exercising after the acute stage of KD [5,6]. Similar results were discovered by a study using the Z-score of CA, which is a reliable scale for detecting CA aneurysms, besides the standard use of exercise tests [7]. However, little has been emphasized on whether gender differences would lead to contrary findings.

It is known that obesity and cardiovascular diseases are positively associated [8,9]. While KD has also been reported to affect the coronary artery, a recent study pointed out that obesity may be an independent risk factor for coronary artery lesions among KD patients [10,11,12]. Therefore, it is important to discuss body mass index (BMI), which is negatively associated with a patient’s exercise capacity, as an indicator of CPF in patients with KD [13]. The existing literature focuses on the correlation between KD patients and their cardiopulmonary function or exercise capacity, or the relationship between BMI and CPF. Therefore, this study aimed to investigate the effect of gender differences on the CPF in KD patients.

## 2. Materials and Methods

### 2.1. Subject Characteristics

This retrospective cohort study was conducted at a tertiary medical center in southern Taiwan. We studied all participants referred to the pediatric cardiology outpatient clinic between July 2012 and July 2021 for a regular follow-up of KD. The patients were included if they had completed a transthoracic echocardiographic examination, standard 12-lead electrocardiogram, and cardiopulmonary exercise testing. The exclusion criteria were: (1) unconfirmed date of onset of KD, (2) patient with under other heart diseases that may affect the cardiopulmonary function (e.g., coronary artery disease not related to KD, moderate to severe cardiac valvular disease, severe arrhythmia, ventricular hypertrophy, or heart failure, (3) known concurrent pulmonary disease or (4) patient’s refusal to participate. We recorded the basic characteristics for all participants, including age, sex, weight, height, BMI, and date of onset of KD. This study was conducted under the Helsinki Declaration; ethical approval was obtained from the Institutional Review Board of Kaohsiung Veterans General Hospital, Taiwan (approval number: VGHKS17-CT11-11).

### 2.2. Anthropometry-Body Composition

A body composition analyzer, Zeus 9.9 PLUS (Jawon Medical Co., Ltd., Kungsang Bukdo, Korea), which utilizes the tetrapolar electrode method, was adapted to analyze the body composition through vector bioelectrical impedance analysis. After entering the participant’s basic data (sex, age, height, weight, and newly calculated body impedance), the analyzer calculated their BMI (body weight in kilograms is divided by squared height in meters). The participants were then categorized into four groups–underweight, normal, overweight, and obese–according to the age and gender-specific reference BMI values suggested by the Ministry of Health and Welfare in Taiwan (2018) [14]. 

### 2.3. Cardiopulmonary Exercise Test (CPET)

CPET was used to measure patients’ cardiopulmonary functions and exercise load capacity performance. We used MasterScreen CPX (CareFusion Germany 234 GmbH, Hochberg, Germany), a combination of standard exercise testing along with ventilatory gas exchange measurement; the testing was done using the ramped Bruce protocol suggested by the American College of Sports Medicine (ACSM). Before beginning the test procedure, the purpose of the test was thoroughly explained to the participant and their caregivers, and consent was obtained (verbally from the participants and in writing from their guardians).

Throughout the test, the participant’s blood pressure, heart rate (HR), respiratory exchange ratio (RER), and minute ventilation (VE) were closely monitored. The oxygen consumption (VO_2_) and carbon dioxide production (VCO_2_) were measured through the breath-to-breath method. The peak VO_2_ was determined when two of the following three conditions were met: (1) RER > 1.1, (2) HR within 5% of the age-predicted maximum, or (3) the participant is exhausted and refused to continue despite strong verbal encouragement [15].To measure the peak metabolic equivalents (METs), the peak VO_2_ level was then divided by 3.5 mLkg^−1^ min^−1^. The anaerobic threshold (AT) was derived via VE/VO_2_ and VE/VCO_2_ methods [16]. The test was terminated when participants showed subjective symptoms, expressed their wish to discontinue, or reached the maximal effort as defined by the ACSM [17]. All CPET trials were performed under the supervision of an experienced physiatrist (K.L.L.) specialized in CPET.

### 2.4. Echocardiography Records

All the echocardiography of each participant was done within one month before or after the date of CPET. Two well-experienced pediatric cardiologists used a sector probe with a more than 5-MHz frequency to do the echocardiography based on the standard measurement methods for pediatric CA recommended by the Japanese Society of Kawasaki Disease [18]. All patients with KD were examined in the supine or right decubitus position and underwent complete 2-dimensional echocardiographic studies with color flow and spectral Doppler examination. Coronary changes were classified using 1984 Japanese Ministry of Health criteria as small (<5 mm), medium (5–6 mm), and giant aneurysms (>6–8 mm) [19].

### 2.5. Statistical Analysis

We used SPSS for Windows version 21.0 (IBM Corp., Armonk, NY, USA) for all analyses. Continuous data were presented as mean ± standard deviation and categorical variables as absolute numbers or percentages. Normality and homoscedasticity were examined before each analysis. To compare data between sexes and participants from various BMI groups, an independent *t*-test was used for normally distributed variables, while the Mann-Whitney U test was used for non-normally distributed variables. A comparison of exercise capacity for each BMI group was performed using the one-way analysis of variance, and the Bonferroni post-hoc test was performed for variance homogeneity. A *p*-value of <0.05 was considered statistically significant.

## 3. Results

A total of 211 participants were found eligible for inclusion into the study; seven of them were later excluded due to incomplete data (BMI or adequate medical record). Hence, the final analysis was done for 204 children (120 boys and 84 girls) who were categorized based on their BMI into underweight (19 boys and 12 girls), normal (62 boys and 59 girls), and overweight (39 boys and 13 girls), respectively.

### 3.1. Demographic Characteristics

Table 1 presents a gender-based comparison between the baseline characteristics. We observed that there was no significant difference between the two gender groups in terms of age (*p* = 0.612), height (*p* = 0.061), and BMI (*p* = 0.072). However, boys weighed significantly more than girls (*p* = 0.012). Surprisingly, there was a significant difference (*p* = 0.029) between the two genders for the normal BMI category, whereas boys had a higher percentage belonging to the overweight group. The percentage of boys with KD who were overweight and obese was higher than girls with KD.

The mean age of all KD patients, girls, and boys with KD were 13.60 ± 5.57, 13.36 ± 5.15, and 13.77 ± 5.86 years old, respectively. The mean duration from diagnosis to the date of CPET of all KD patients, girls, and boys with KD were 10.51 ± 6.24, 10.06 ± 6.66, and 11.12 ± 5.60 years, respectively. Four (4.8%) girls and six (5.0%) boys presented with CA aneurysm at the time of receiving CPET. Among the participants with CA aneurysm, one boy with normal BMI presented with a giant one, one girl with normal BMI presented with a medium one, and the others all presented with a small one.

### 3.2. Comparison of Cardiopulmonary Fitness among Subjects with Various BMIs

Table 2 summarizes the comparison of CPF among subjects with various BMIs through the post-hoc Bonferroni analysis. Irrespective of the gender, the mean peak RER values exceeded 1.1 (*p* = 0.277) for all BMI groups (the overweight group was placed with the obese group), suggesting that maximal oxygen exercise efforts were reached. In AT, both VO_2_ and peak VO_2_ represent absolute oxygen consumption (mL/min), where the heavier in weight the greater the number. The BMI-based group-wise analysis showed significantly different values for AT VO_2_ (*p* < 0.001) and nearly different values for peak VO_2_ (*p* = 0.098) for all groups, which is comparable to the values for normal children.

AT MET and peak MET are relative values (mL/min/kg) that indicate aerobic capacity, so the higher the number the better capacity. We found significantly differences in AT MET (*p* = 0.001) and peak MET (*p* = 0.020) values between the different BMI groups.

The peak oxygen consumption to predicted value (peak PD) is a relative value is described as a percentage compared with normal peers, in which 100% is normal and at least >80% is desired. We observed that peak PD for the overweight and obese participants was significantly worse than the normal and underweight group (*p* < 0.001).

On analyzing the KD boys with different BMIs, we found that both the absolute and relative oxygen consumptions showed significant differences for AT VO_2_ (*p* = 0.015), peak VO_2_ (*p* = 0.047), AT MET (*p* < 0.001), peak MET (*p* < 0.001), and peak PD (*p* < 0.001), indicating that greater BMI led to poorer exercise capacity in boys.

On the contrary, the analysis for KD girls found a significant difference in the absolute oxygen consumption values (AT VO_2_, *p* = 0.006; peak VO_2_, *p* = 0.002), but not for the relative oxygen consumption (AT MET, *p* = 0.417; peak MET, *p* = 0.818), and peak PD (*p* = 0.268). Although the absolute oxygen consumption had significant differences, the relative oxygen consumption indicated an equivalent exercise capacity for different BMIs.

### 3.3. Comparison of Cardiopulmonary Fitness between Girls and Boys with Various BMIs

Table 2 also demonstrates the comparison of cardiopulmonary fitness between girls and boys with various BMIs through independent *t*-test. In respect of total and normal BMI groups, KD boys had higher AT MET, AT VO_2_, peak MET, and peak VO_2_ than KD girls (*p* values from <0.001 to 0.012). In respect of underweight and overweight/obesity groups, both boys and girls had comparable AT MET, AT VO_2_, peak MET, and peak VO_2_ except that underweight KD boys had higher peak MET than girls (*p* = 0.009). As for peak PD, KD girls had higher significant values than KD boys regardless of BMI group (*p* values from <0.001 to 0.017).

## 4. Discussion

Our study findings resonate with some existing research while offering some novel insights. We found that girls with KD have better CPF than KD boys. We believe that the gender stereotype effect on sport participation as well as self -efficacy might be a determinant for KD boys having poorer CPF than KD girls. 

On comparing the participants’ BMIs in light of different genders, we noticed a significant discrepancy for the overweight/obese groups. As revealed in an overall analysis of childhood and adolescent obesity conducted by the Taiwan National Council on Physical Fitness and Sports, a similar percentage for BMI contribution in overweight/obese girls (16.5%) was found compared with our KD girls (15.4%). On the other hand, the BMI distribution for Taiwanese boys, in general (26.8%), was much different from our KD boys (32.5%) [20]. Therefore, the positive association between KD and overweight/obesity boys is indicated.

Regarding the correlation between BMI and CPF, boys with normal weight had significantly better CPF than the overweight and obese ones. This finding is in line with a previous study that revealed that non-obese children have better CPF than overweight/obese children [21].

One study showed that children and adolescents with KD could maintain normal CPF after the disease’s acute stage has subsided [6]. Therefore, it was necessary to evaluate exercise capacity between the two sexes through relative oxygen consumption values. Besides, relative oxygen consumption values are crucial for knowing whether AT VO_2_, peak VO_2_ values would increase along with the increase of BMI. Our data showed that AT MET, peak MET, and peak PD values for Taiwanese boys were considerably lower than girls, which suggests a better CPF in KD girls than KD boys.

Another interpretation of our study result is that KD possibly affects boys more than girls physically as well as mentally. Parents of children with chronic illnesses, such as KD, often experience anxiety or fear about the uncertain sequela of coronary artery involvement or the unpredictability of disease progression [22]. It is known that the patients and their parents suffer from the fear of bleeding related to trauma troubled when they are taking anticoagulation medications [22]. Therefore, lack of physical activity could increase BMI, result in poor CPF, and increase health risks.

Previous nationwide studies were conducted using questionnaires or interviews that discussed how an illness would affect a child and their surrounding people. For instance, self-perceptions were believed to be reduced in patients with acute lymphoblastic leukemia, and mastery experiences might play a big role in enhancing self-perceptions toward physical activity [23]. Self-efficacy was then brought into light for patients with congenital heart disease who are generally recommended limited physical activity by a cardiologist, leading to greater anxiety in the parents. A few studies have reported that persuasion from cardiologists and mothers greatly influenced self-efficacy toward physical activity in children with heart disease [24,25]. Further, when talking about the defiance of physical capacity between the two genders of congenital heart disease patients, girls showed a more vigorous resistance than the boys significantly [26]. While there is no reported significant association between disease severity and physical activity, self-efficacy was found to override the severity of the cardiac problem in determining the performance [27]. In addition, gender stereotypes on sport participation might be the main factor contributing to the discrepancy of peak VO_2_ between male and female KD subjects. It has been stated that cultural stereotyping of sport participation regarding physical strength and prowess, body image, and opportunities for team support limited the choices for male subjects, and therefore, may contribute to their lower sport-related self-efficacy than for female subjects with congenital heart disease [24,26]. Regarding the KD patients, a strong correlation was seen between the amount of weekly exercise and exercise motivation and self-efficacy [28].

This study had certain limitations. The study was conducted in a single medical center in southern Taiwan, so the results may be suitable for generalizing to specific populations. Further, the patients with relatively severe Z-score were excluded from this study; however, the KD population and composition are known to be affected by different severity in each gender. In addition, there were only 13 overweight/obese girl participants included in our study. Therefore, it is plausible that the relatively small sample size comparing to overweight/obese boy group resulting in statistical bias to some extent. Finally, there was insufficient data concerning gender differences through Taiwanese social and cultural perspectives, or the qualitative survey of physical activities engaged by participants included in this study. Future investigations should be designed as prospective cohort studies through questionnaires or interviews to acquire a complete understanding of the relationship regarding a Taiwanese participant’s physical condition and psychological expectations.

## 5. Conclusions

Girls with KD had better relative oxygen consumption values in terms of AT MET, peak MET, and peak PD, which means their CPF is better than boys. Surprisingly, there was a significant difference between the various BMI categories where boys had a higher percentage in the overweight group. Therefore, the association between KD and obesity, in addition to gender stereotypes and self-efficacy, may contribute to better CPF in KD girls. Nevertheless, there is a need for more investigations for further evaluation.

## Figures and Tables

**Table 1:** Baseline characteristics of all children with Kawasaki disease.

	Age (Years)	Height (cm)	Weight (kg)	BMI (kg/m^2^)	U (%)	N (%)	O (%)	F (%)	KD Duration (Years)	CA Aneurysm N(%)
Girl (N = 84)	13.36 ± 5.15	147.39 ± 16.38	43.95 ± 14.62	19.57 ± 3.70	13.3	71.1	10.8	4.8	10.06 ± 6.66	4 (4.8)
Boy (N = 120)	13.77 ± 5.86	152.53 ± 20.76	50.25 ± 20.58	20.64 ± 4.67	15.8	51.7	25.0	7.5	11.12 ± 5.60	6 (5.0)
Total (N = 204)	13.60 ± 5.57	150.43 ± 19.21	47.67 ± 18.60	20.20 ± 4.32	14.8	59.6	19.2	6.4	10.51 ± 6.24	10 (4.9)
*p* value ^a^	0.612	0.061	0.012 *	0.072		0.029 *			0.250	0.886

BMI: body mass index; U (%), percentage of underweight subjects; N (%), percentage of normal weight subjects; O(%), percentage of overweight subjects; F (%), percentage of obesity subjects; CA aneurysm N(%), number and (percentage) of subjects presented with coronary artery aneurysm. * *p* < 0.05. ^a^ All the comparisons between girls and boys were done by independent t test except *p* values that marked with ^a^, which was analyzed by independent Chi square test for comparison percentage of excessive adiposity between girls and boys.

**Table 2:** Comparisons of cardiopulmonary fitness between subjects with underweight, normal, and overweight body mass index.

	AT MET	AT VO_2_	Peak MET	Peak VO_2_	Peak RER	Peak PD
Total (N = 204)	6.67 ± 1.41	1072.90 ± 400.47	9.71 ± 1.97	1573.44 ± 583.84	1.16 ± 0.10	75.20 ± 15.37
Underweight (n = 31)	7.15 ± 1.46	909.30 ± 355.39	10.52 ± 2.04	1360.59 ± 504.49	1.14 ± 0.08	81.25 ± 14.77
Normal (n = 121)	6.80 ± 1.37	1030.44 ± 371.03	9.87 ± 1.93	1507.59 ± 565.24	1.17 ± 0.01	77.66 ± 14.00
Overweight/Obesity (n = 52)	6.08 ± 1.31	1269.24 ± 424.63	8.88 ± 1.74	1849.45 ± 582.26	1.17 ± 0.11	65.88 ± 15.03
*p* value	0.001 *^,a,b^	<0.001 *^,a,b^	0.020 *^,a,b^	0.098	0.277	<0.001 *^,a,b^
Boys (N = 120)	6.87 ± 1.45	1155.07 ± 424.18	10.19 ± 2.04	1716.32 ± 612.19	1.16 ± 1.00	69.21 ± 13.07
Underweight (n = 19)	7.39 ± 1.37	954 ± 353.15	11.24 ± 2.04	1455.24 ± 513.66	1.14 ± 0.07	75.72 ± 12.13
Normal (n = 62)	7.21 ± 1.27	1133.25 ± 412.19	10.69 ± 1.69	1697.78 ± 634.76	1.17 ± 0.10	72.87 ± 11.06
Overweight/Obesity (n = 39)	6.08 ± 1.47	128.60 ± 438.96	8.88 ± 1.92	1873.00 ± 584.84	1.17 ± 0.12	60.24 ± 11.97
*p* value	<0.001 *^,a,b^	0.015 *^,a^	<0.001 *^,a,b^	0.047 *^,a^	0.506	<0.001 *^,a,b^
Girls (N = 84)	6.39 ± 1.32	955.52 ± 322.52	9.03 ± 1.64	1366.86 ± 471.95	1.17 ± 0.10	83.75 ± 14.39
Underweight (n = 12)	6.78 ± 1.57	838.15 ± 362.45	9.29 ± 1.41	1197.08 ± 465.52	1.14 ± 0.87	87.80 ± 13.18
Normal (n = 59)	6.37 ± 1.36	922.40 ± 288.03	9.01 ± 1.79	1307.74 ± 397.43	1.17 ± 0.10	82.69 ± 15.06
Overweight/Obesity (n = 13)	6.08 ± 0.74	1214.15 ± 389.55	8.87 ± 1.11	1778.83 ± 592.06	1.17 ± 0.08	82.81 ± 9.61
*p* value	0.417	0.006 *^,a,b^	0.818	0.002 *^,a,b^	0.557	0.268
Between Sexes comparison (*p* value)	AT MET	AT VO_2_	Peak MET	Peak VO_2_	Peak RER	Peak PD
Total	0.012 *	<0.001 *	<0.001 *	<0.001 *	0.817	<0.001 *
Underweight	0.192	0.486	0.009 *	0.181	0.983	0.017 *
Normal	0.001 *	0.001 *	<0.001 *	<0.001 *	0.853	<0.001 *
Overweight/Obesity	0.99	0.594	0.982	0.618	0.936	<0.001

AT MET, metabolic equivalent at anaerobic threshold; Peak MET, peak metabolic equivalent during exercise testing; AT VO_2_, oxygen consumption at anaerobic threshold; Peak VO_2_, peak oxygen consumption during exercise testing; RER, respiratory exchange ratio; peak PD, percentage of measured peak oxygen consumption to predicted value. * *p* value < 0.05. Post-Hoc analysis by Bonferroni test found significant difference between ^a^ overweight and underweight, ^b^ overweight and normal.

## Data Availability

Data sharing statement: Individual participant data that underlie the results reported in this article, after deidentification might be shared. Proposals should be directed to Gabbrile@vghks.gov.tw. for application. (accessed on 23 September 2021).
